# Lower frequency of T stem cell memory (TSCM) cells in hepatitis B vaccine nonresponders

**DOI:** 10.1007/s12026-022-09278-9

**Published:** 2022-04-20

**Authors:** Mahsa Eshkevar Vakili, Zahra Faghih, Jamal Sarvari, Mehrnoosh Doroudchi, Seyed Nezamedin Hosseini, Dieter Kabelitz, Kurosh Kalantar

**Affiliations:** 1 Department of Immunology, School of Medicine, Shiraz University of Medical Sciences, Shiraz, Iran; 2 School of Medicine, Shiraz Institute for Cancer Research, Shiraz University of Medical Sciences, Shiraz, Iran; 3 Department of Bacteriology and Virology, School of Medicine, Shiraz University of Medical Sciences, Shiraz, Iran; 4 Gastroenterohepatology Research Center, Shiraz University of Medical Sciences, Shiraz, Iran; 5 Department of Recombinant Hepatitis B Vaccine, Production and Research Complex, Pasteur Institute of Iran, Tehran, Iran; 6 Institute of Immunology, Christian-Albrechts University of Kiel and University Hospital Schleswig, Holstein Campus Kiel, 24105 Kiel, Germany

**Keywords:** Hepatitis B vaccine, CD4^+^ memory T cells, Memory T cells producing IFN-γ, T stem cell memory, T central memory, T effector memory

## Abstract

Despite the availability of an effective vaccine and antiviral treatments, hepatitis B is still a global public health problem. Hepatitis B vaccination can prevent the disease. Vaccination induces long-lasting protective immune memory, and the identification of memory cell subsets can indicate the effectiveness of vaccines. Here, we compared the frequency of CD4^+^ memory T cell subsets between responders and nonresponders to HB vaccination. Besides, the frequency of IFN-γ^+^ memory T cells was compared between studied groups. Study participants were grouped according to their anti-HBsAb titer. For restimulation of CD4^+^ memory T cells, peripheral blood mononuclear cells (PBMCs) were cultured in the presence of HBsAg and PHA for 48 h. Besides, PMA, ionomycin, and brefeldin were added during the last 5 h of incubation to induce IFN-γ production. Flow cytometry was used for analysis. There was a statistically significant difference in the frequency of CD4^+^CD95^+^, CD4^+^CD95^Hi^, and CD4^+^CD95^low/med^ T stem cell memory (T_SCM_) cells between responder and nonresponder groups. However, the comparison of the frequency of memory T cells producing IFN-γ showed no differences. Our results identified a possible defect of immunological CD4^+^ memory T cell formation in nonresponders due to their lower frequency of CD4^+^ T_SCM_ cells.

**Supplementary Information:**

The online version contains supplementary material available at 10.1007/s12026-022-09278-9.

## Introduction

Hepatitis is a term used for a variety of inflammatory liver diseases which may eventually lead to liver failure, cirrhosis, and hepatocellular carcinoma. This inflammation is divided into two main categories: noninfectious and infectious, which is induced by hepatitis A, B, C, D, and E viruses as well as cytomegalovirus, and Epstein–Barr virus [1]. Hepatitis B and C viruses are the major causes of cirrhosis and liver cancer [2, 3]. Hepatitis B virus (HBV), a member of the *Hepadnaviridae* family [4], has a small, partially double-stranded, relaxed-circular DNA genome that encodes seven proteins: precore/E antigen (HBeAg), large (L-), medium (M-), and small (S-) surface antigen (HBsAg), core protein, polymerase, and X protein (HBx) [5, 6]. Nowadays, it is well known that hepatocyte infection with the virus is non-cytopathic and can be transient or chronic depending on the ability of the host immune system to clear the infection [7].

Despite the availability of effective vaccines and antiviral treatments, infection with HBV is considered a global health problem [8, 9]. In the past 2 decades, many drugs have been developed to treat this disease. Their main problems are the inability to eradicate HBV, side effects, the necessity for regular injections, and the high cost of treatment. In addition to the suggested treatments, hepatitis B (HB) vaccination seems to be the most effective strategy to prevent and control the infection [10–14]. The first generation of the HBV vaccine was the serum of people who produced large amounts of antibodies against HBsAg (passive). Subsequently, advances in DNA recombination technology led to the development of the second generation of HBV vaccines (DNA recombinant vaccines) (active) [15, 16].

After vaccination, measurement of the humoral immune response against HBsAg is an immune marker indicating the presence or absence of protective antibodies against HBV infection. According to this factor, seroprotection is accepted when anti-HBsAb titer reaches more than 10 mIU/ml, therefore, people who did not develop corresponding anti-HBsAb titers, even after administration of two complete series of the HBV vaccine, are considered nonresponders [15, 17, 18].

After HBV exposure, protection induced by the vaccine occurs through two mechanisms; the first is the neutralization of the virus by anti-HB antibodies, and the second involves the activation of CD4^+^ T memory cells, which subsequently activate memory B cells to secrete anti-HBs antibody [15]. Several studies have shown that vaccine-induced antibody levels are gradually declining, while memory cell formation in healthy recipients will remain for more than 15 years [19–22].

Although, many successful vaccines primarily act by generating antibodies, producing vaccines that can provoke a population of highly-specific T cells is completely on demand. These types of vaccines should have the ability to generate large, effective, and long-lived populations of memory T cells [23, 24]. Advances in the multi-parameter flow cytometry technique have provided the ability to define the heterogeneity of T cells [25]. Based on the differential expression of CD28, CCR7, CD45RO, and CD95, there are six major groups of quiescent T cells, including naïve T cell (T_N_), stem cell memory T cell (T_SCM_), central memory T cell (T_CM_), transitional memory T cell (T_TM_), effector memory T cell (T_EM_), and terminal effector T cell (T_TE_). These cells are supposed to be generated from T_N_ during a linear model called linear differentiation [23, 26–29]. Despite many advances in this field, there are still questions about the formation and maintenance of immunological memory after vaccination [23]. Accordingly, the identification of memory cell subsets can indicate the effectiveness of vaccines like the HB vaccine. In the present study, we, therefore, aimed to determine the frequency of CD4^+^ memory T cell subsets and compare these cell quantities between responders and nonresponders to the HB vaccine.

## Materials and methods

### Subjects

All study participants were selected from the health care staff of hospitals affiliated with Shiraz University of Medical Sciences (SUMS), Shiraz, Iran. This study was approved by the Ethics Committee of the university (ethics code: IR.SUMS.REC.1397.779).

The participants were divided into two groups of responders (*n* = 13) and nonresponders (*n* = 15) according to their anti-HBsAb titers registered at the hospital infection control centers. Responders had antibody titers > 100 mIU/ml, and nonresponder subjects had antibody titers < 10 mIU/ml after administration of at least two complete series of the HB vaccine (repeating the whole schedule of vaccine after the first schedule). After obtaining written informed consent, the blood samples were taken under sterile conditions and transferred to the laboratory enclosed in an ice pack. The patients with hepatic infections and HIV, cancers, autoimmune diseases, and alcohol users were excluded from the study.

### Isolation of mononuclear cells from peripheral blood

Five milliliters of peripheral blood were overlaid on Ficoll-Paque (inno-train DIAGNOSTIK GMBH, Germany) in sterile conditions to isolate mononuclear cells by density gradient centrifugation. The plasma layer was removed, aliquoted, and sorted at − 20 °C for identification of anti-HBs Ab level. The peripheral blood mononuclear cells (PBMCs) were then carefully aspirated from the Ficoll-plasma interface, washed two times with 1 × phosphate-buffered saline (PBS), counted with Trypan blue dye (Shellmax, China), and prepared in appropriate concentration for further studies.

### Determination of HBsAb with enzyme linked immunoassay (ELISA) technique

Anti-HBsAb plasma level was checked using an HBsAb ELISA kit (DIA.PRO Diagnostic Bioprobes Srl, Italy) according to the manufacturer’s instructions. In brief, the sample was applied on microwells coated with highly purified HBsAg, which specifically captured anti-HBs antibodies and formed antibody‐antigen complexes. The amount of conjugate bound and, hence, the color in the wells was directly related to the concentration of antibodies in the sample.

### Activation for restimulation of CD4^+^ memory T cells

Two million of PBMCs were cultured in a final volume of 1000 µl complete culture media (CM10) [RPMI 1640 containing 10% heat-inactivated fetal bovine serum (FBS), 1% penicillin–streptomycin (Pen-Strep), and 1% glutamine (all from Shellmax)] per well. The optimal concentrations of HBsAg (Razi Institute, Iran) and phytohemagglutinin (PHA; Invitrogen, USA) were determined to be 4 and 1 µg/ml for lymphocyte activation. The cells were then exposed to HBsAg and PHA for 48 h at 37 °C in a humidified atmosphere supplemented with 5% CO_2_.

### Activation for IFN-γ production by CD4^+^ T cells

Two million of PBMCs were cultured in a final volume of 1000 µl CM10 per well. The cells were stimulated with HBsAg (4 µg/ml) (Razi Institute) and PHA (1 µg/ml) (Invitrogen) during a 48 h incubation at 37 °C in a humidified atmosphere supplemented with 5% CO_2_. In the last 5 h, 25 ng/ml of phorbol myristate acetate, 500 ng/ml of ionomycin (both from Sigma-Aldrich, USA), and 0.7 µl of brefeldin A as a Golgi stopper (BD Biosciences, USA) were added to this cocktail.

### Cell staining for assessment of CD4^+^ memory T cell subsets

As previously described [30], to investigate the frequency of CD4^+^ memory T cell subsets, after stimulation time, PBMCs were collected, washed with 1 × PBS, and stained with appropriate fluorescent-labeled antibodies (FITC-conjugated anti-CCR7 clone: G043H7, PE-conjugated anti-CD95 clone: Dx2, PerCP/Cy5.5-conjugated anti-CD4 clone: RPA-T4, and APC-conjugated anti-CD45RO clone: UCHL1; all from Biolegend, USA) and incubated in the dark for 30 min at room temperature. After that, the cells were washed twice using 2 ml of 1 × PBS to remove unbound antibodies and fixed in 300 µl of paraformaldehyde (PFA; 10 mg/ml; Merk, Germany) for 15 min at 4 °C. Following washing with 3 ml of 1 × PBS, in the last step, the cells were suspended in 500 µl of 1 × PBS and acquired on a 4-color BD FACSCalibur™ flow cytometer (BD Biosciences) (~ 200 × 10^3^ events). FlowJo software (version X.0.7; Tree Star, Inc., Ashland, OR, USA) was used for data analysis.

The mean fluorescent intensity (MFI) of CD95 was also evaluated on CD95^+^ and CD95^Hi^ T_SCM_. To normalize the MFI of CD95 in different subsets, the MFI of CD95 in positive cells (CD95^+^ T_SCM_ or CD95^Hi^ T_SCM_) was divided by the MFI of CD95 in the negative population (T_N_).

### Cell staining for assessment of CD4^+^ IFN-γ^+^ memory T cells

At the end of stimulation time, PBMCs were collected, washed with 1 × PBS, and stained with fluorescent-labeled antibodies for both surface and intracellular markers. At first, an APC-conjugated anti-CD45RO antibody (clone: UCHL1; Biolegend) was added, and the cells were incubated in the dark for 30 min at room temperature. Then, they were washed twice with 2 ml of 1 × PBS and fixed with 300 µl of PFA (10 mg/ml; Merk) for 15 min at 4 °C. After washing with 3 ml of 1 × PBS, in the next step, the cells were permeabilized using 1 ml of 1 × Perm/Wash buffer (Biolegend) and were incubated in the dark for 15 min at room temperature. The fluorescent-labeled antibodies (FITC-conjugated anti-IFN-γ clone: B27 and PerCP/Cy5.5-conjugated anti-CD4 clone: RPA-T4; Biolegend) were then added, and incubation was done in the dark for 30 min at room temperature. The cells were washed twice with 1 × Perm/Wash buffer and then fixed with 300 µl of PFA for 15 min at 4 °C. Finally, the cells were washed with 3 ml of 1 × PBS, suspended in 500 µl of 1 × PBS, and were acquired on a 4-color BD FACSCalibur™ flow cytometer (BD Biosciences) (~ 200 × 10^3^ events). FlowJo software (version X.0.7; Tree Star, Inc.) was used for data analysis.

As the mean expression of IFN-γ, the MFI of this cytokine was also determined. To report the MFI of IFN-γ, the MFI of each IFN-γ expressing T cell was normalized by the corresponding IFN-γ negative population.

### Statistical analysis

SPSS software (version 22.0; IBM Corp., Armonk, NY, USA) was used for statistical analysis. Before comparing, the normal distribution of variables was first evaluated using the Kolmogorov–Smirnov test. As the data could not pass the normality test, the nonparametric Mann–Whitney *U* test was used. Moreover, a nonparametric Spearman’s rank correlation test was done to assess the relationship between two quantitative variables. All data were presented as mean ± SEM, and *p*-values < 0.05 were considered significant.

## Results

### The characteristics of the study subjects

In this study, 15 people with anti-HBs antibody titer less than 10 mIU/ml were recruited as the nonresponder group. These individuals had no increase in their anti-HBsAb titer after administration of at least 2 complete series of the HBV vaccine. Besides, 13 subjects with an antibody titer of more than 100 mIU/ml were included in the responder group (Table 1). The individuals with inflammatory diseases (i.e., autoimmune disorders and cancers), alcohol users, and those infected by HIV, HBV, and HCV were excluded from the investigation.

**Table 1:** The characteristics of the studied subjects

Characteristic		Nonresponders (*n* = 15)	Responders (*n* = 13)
Age (year) (Mean ± SEM)		45.13 ± 2.24	41.15 ± 2.62
Sex *F* (%)	Male	2 (13.34%)	2 (15.38%)
	Female	13 (86.66%)	11 (84.62%)
Anti-HBs Ab titer (mIU/ml) (mean ± SEM)		4.19 ± 1.22	240.02 ± 7.46

*F*, frequency; *SEM*, standard error of mean; *mIU/ml*, milli international unit/milliliter.

### The frequency of different CD4^+^ memory T cell subsets in the peripheral blood of HB vaccine responders and nonresponders after stimulation with HBsAg

**Fig. 1:**
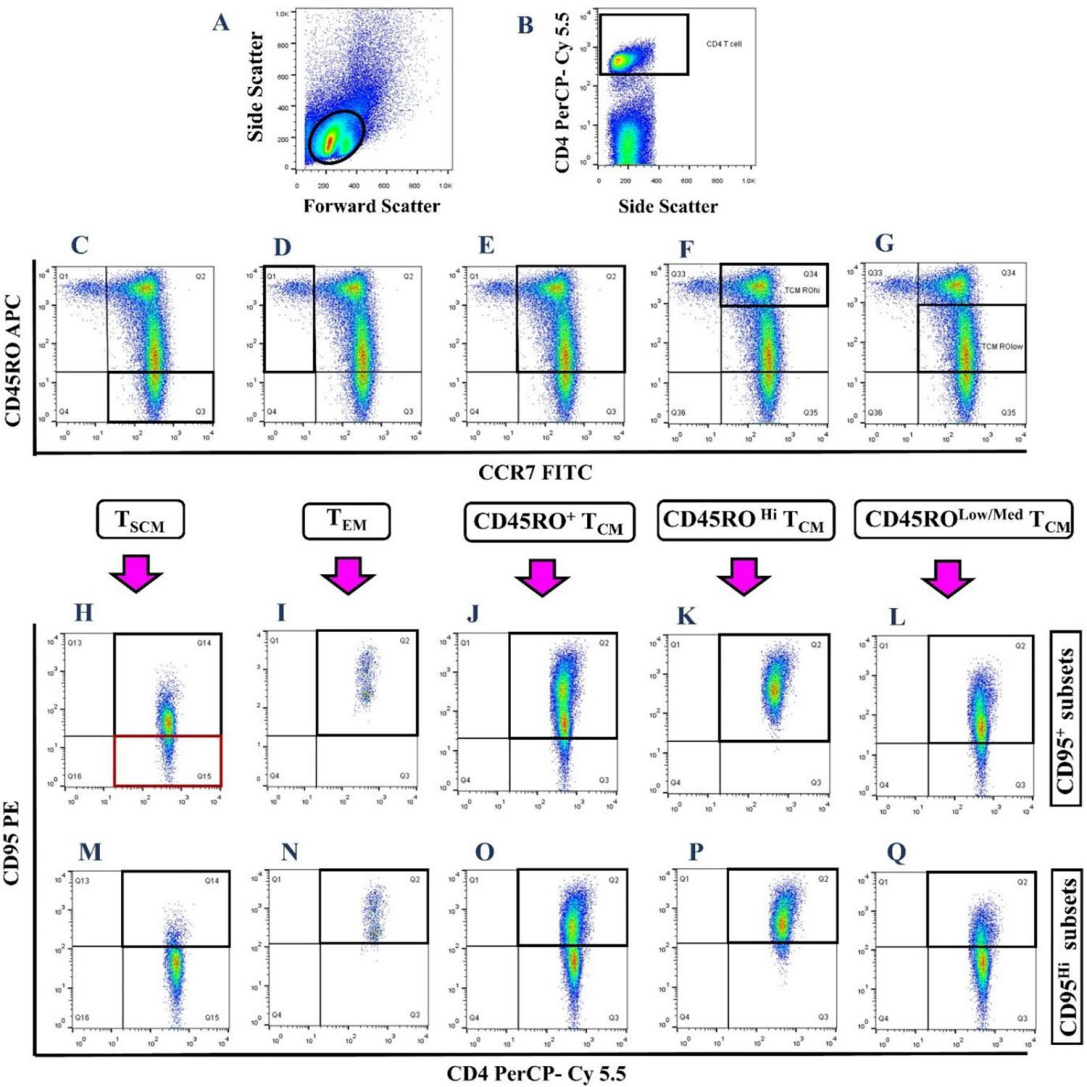
Gating strategy to identify the frequency of CD4^+^ memory T cell subsets in peripheral blood of HB vaccine responders and nonresponders after stimulation. Lymphocytes were gated based on their relative size (forward scatter) and granularity (side scatter) (**A**). Then, lymphocytes with high expression of CD4 (CD4^+^) were determined (**B**). The frequencies of different memory T cell subsets were then defined based on the expression of CCR7, CD45RO, and CD95 markers in the CD4^+^ population (**C–Q**). Regarding different levels of CD95 expression on memory T cell subsets, two groups of cells, CD95^+^ and CD95^Hi^ cells, were evaluated (**H–Q**). T_N_: T naïve; T_SCM_: T stem cell memory; T_CM_: T central memory; T_EM_: T effector memory

Our data analysis in flow cytometry relied on the following gating strategy: lymphocytes were gated based on their relative size (forward scatter) and granularity (side scatter) (Fig. 1A). Then, lymphocytes with high expression of CD4 marker (CD4^+^) were separated (Fig. 1B), and the frequency of different memory T cell subsets was determined based on the expression of CCR7, CD45RO, and CD95 markers. All frequencies were reported in the CD4^+^ T cell population. Among CCR7^+^CD45RO^−^ population (Fig. 1C), CD95^−^ cells were considered T_N_ cells, and those with CD95 expression were introduced as T_SCM_. T_N_ and T_SCM_ cells were determined in red and black squares, respectively, in Fig. 1H. CCR7^+^CD45RO^+^ cells (Fig. 1E) expressing CD95 were considered T_CM_ cells (Fig. 1J). Besides, according to the expression level of CD45RO (Fig. 1F and G), two groups of T_CM_ were further introduced: CD45RO^Hi^ T_CM_ (Fig. 1K) and CD45RO^low/med^ T_CM_ (Fig. 1L). The CCR7^−^CD45RO^+^ cells (Fig. 1D) expressing CD95 were considered T_EM_ (Fig. 1I). Regarding the high expression of CD95 on memory T cell subsets after activation, CD95^Hi^ memory T cells were also evaluated (Fig. 1M-Q). The frequency of CD95^low/med^ memory T cells was also calculated by subtracting the frequency of CD95^Hi^ memory T cell from the frequency of CD95^+^ memory T cell.

### Frequency of CD4^+^ memory T cell subsets in responders and nonresponders to HB vaccine

The purpose of this part of the experiment was to determine the differences in the frequency of the CD4^+^ memory T cell subsets between the responder and nonresponder groups. Median (IQ25–75) and mean ± SEM of the frequency of cell subsets in each group and *p*-values of their differences were detailed in Table 2. As shown, there were no statistically significant differences in the frequencies of various memory T cell subsets except for CD4^+^CD95^+^ (*P*-value = 0.023), CD4^+^CD95^Hi^ (*P*-value = 0.001), and CD4^+^CD95^low/med^ (*P*-value = 0.032) T_SCM_ cells between responder and nonresponder groups. The CD95 expression (based on MFI) on CD4^+^ T_SCM_ was also compared, however, there were no statistical significant differences between the two groups (Table 2). The results obtained from responder and nonresponder groups were shown in Supplementary Tables S1 and S2, respectively.

**Table 2:** Frequencies of CD4^+^ memory T cell subsets between responders and nonresponders to HB vaccine

Subset	Phenotype	Nonresponder		Responder		*P*-value
		**Median** **(IQ25–75)**	**Mean ± SEM**	**Median** **(IQ25–75)**	**Mean ± SEM**	
**T**_**N**_	CD4^+^CCR7^+^CD45RO^−^CD95^−^	5.23 (3.09–7.36)	5.79 ± 0.92	3.92 (3.08–7.18)	5.46 ± 0.92	0.836
**T**_**SCM**_	CD4^+^CCR7^+^CD45RO^−^CD95^+^	12.92 (8–17.84)	13.37 ± 1.45	19.01 (13.78–23.07)	19.59 ± 1.78	**0.023**
	CD4^+^CCR7^+^CD45RO^−^CD95^Hi^	1.05 (0.67–1.39)	1.08 ± 0.14	1.87 (1.46–2.47)	2.02 ± 0.19	**0.001**
	CD4^+^CCR7^+^CD45RO^−^CD95^low/med^	12.07 (7.37v16.63)	12.28 ± 1.34	17.13 (12.17–20.36)	17.57 ± 1.63	**0.032**
**T**_**CM**_	CD4^+^CCR7^+^CD45RO^+^CD95^+^	68.25 (59.44–76.44)	67.93 ± 2.37	66.28 (56.19–71.92)	65.73 ± 2.47	0.596
	CD4^+^CCR7^+^CD45RO^+^CD95^Hi^	38.25 (31.26–47.75)	40.38 ± 2.48	36.58 (31.37–39.72)	37.09 ± 2.19	0.345
	CD4^+^CCR7^+^CD45RO^+^CD95^low/med^	23.98 (19.84–36.63)	27.55 ± 2.58	26.23 (21.74–36.32)	28.64 ± 2.39	0.662
**T**_**CM**_	CD4^+^CCR7^+^CD45RO^Hi^CD95^+^	30.9 (22.68–42.78)	32.75 ± 2.61	27.8 (21.34–33.58)	27.61 ± 2.52	0.189
	CD4^+^CCR7^+^CD45RO^Hi^CD95^Hi^	26.75 (19.98–39.32)	28.26 ± 2.34	26.33 (17.35–27.73)	24.63 ± 2.45	0.369
	CD4^+^CCR7^+^CD45RO^Hi^CD95^low/med^	2.7 (1.14–5.45)	4.49 ± 1.3	2.11 (1.12–4.42)	2.98 ± 0.071	0.596
**T**_**CM**_	CD4^+^CCR7^+^CD45RO^low/med^CD95^+^	36.04 (29.52–41.77)	35.64 ± 1.83	36.05 (30.73–46.1)	38.05 ± 2.36	0.475
	CD4^+^CCR7^+^CD45RO^low/med^CD95^Hi^	12.38 (9.1–14.95)	12.25 ± 0.92	12.66 (10.39–15.55)	12.64 ± 0.93	0.695
	CD4^+^CCR7^+^CD45RO^low/med^CD95^low/med^	22.99 (19.28–27.94)	23.39 ± 1.89	24.41 (19.68–32.6)	25.41 ± 2.09	0.475
**T**_**EM**_	CD4^+^CCR7^−^CD45RO^+^CD95^+^	5.38 (3.3–8.9)	6.8 ± 1.4	2.96 (2.33–5.91)	3.9 ± 0.59	0.069
	CD4^+^CCR7^−^CD45RO^+^CD95^Hi^	3.62 (1.95–5.97)	5.43 ± 1.4	2.8 (1.47–4.31)	2.96 ± 0.5	0.222
	CD4^+^CCR7^−^CD45RO^+^CD95^low/med^	0.8 (0.36–2.18)	1.37 ± 0.38	0.68 (0.16–1.13)	0.94 ± 0.34	0.323
**Mean expression of CD95 on CD4**^**+**^** T**_**SCM**_** cell subsets (based on MFI)**						
**T**_**SCM**_	CD4^+^CCR7^+^CD45RO^−^CD95^+^	3.41 (3.1–5.29)	4.4 ± 0.65	4.48 (3.5–5.7)	4.47 ± 0.34	0.222
	CD4^+^CCR7^+^CD45RO^−^CD95^Hi^	11.31 (8.43–21.28)	16.06 ± 3.72	11.96 (9.56–19.68)	14.05 ± 1.56	0.629

*IQ*, interquartile; *SEM*, standard error of mean; *T*_*N*_, T naïve; *T*_*SCM*_, T stem cell memory; *T*_*CM*_, T central memory; *T*_*EM*_, T effector memory.

### Correlation of anti-HBsAb level and age of participants with the frequencies of different cell subsets

**Fig. 2:**
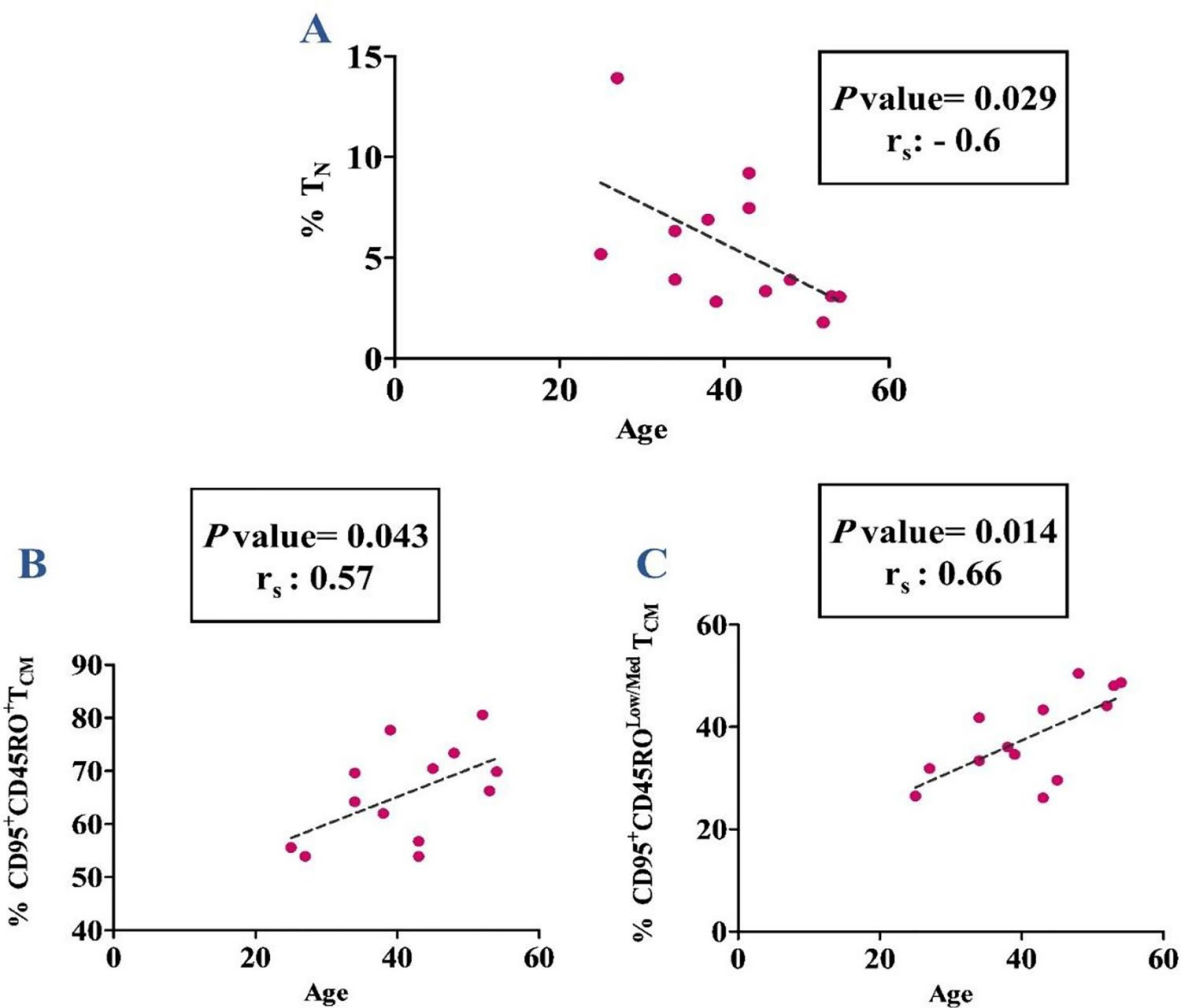
Correlations between age and the frequency of CD4^+^ T_N_ (**A**) and T_CM_ cell subsets (**B** and **C**) in responders to HB vaccine. The frequencies were reported in CD4^+^ lymphocyte population. T_N_: T naïve; T_CM_: T central memory

Our results showed no correlation between the anti-HBsAb titer and the frequencies of various CD4^+^ memory T cell subsets in both responder and nonresponder groups. Moreover, there was no correlation between the age of nonresponders and the percentages of different cell subsets, while a negative correlation was observed between the age of responders and the frequency of CD4^+^ T_N_ (*P*-value = 0.029, rs =  − 0.6). On the other hand, the positive correlations were found between the responders’ age and the frequencies of CD4^+^ T_CM_ subsets (CD45RO^+^ T_CM_: *P*-value = 0.043, rs: 0.57; and CD45RO^low/med^ T_CM_: *P*-value = 0.014, rs = 0.66) (Fig. 2, Table S3).

### Production of IFN-γ by CD4^+^and CD4^−^ memory T cells of responders and nonresponders to HB vaccine after stimulation

**Fig. 3:**
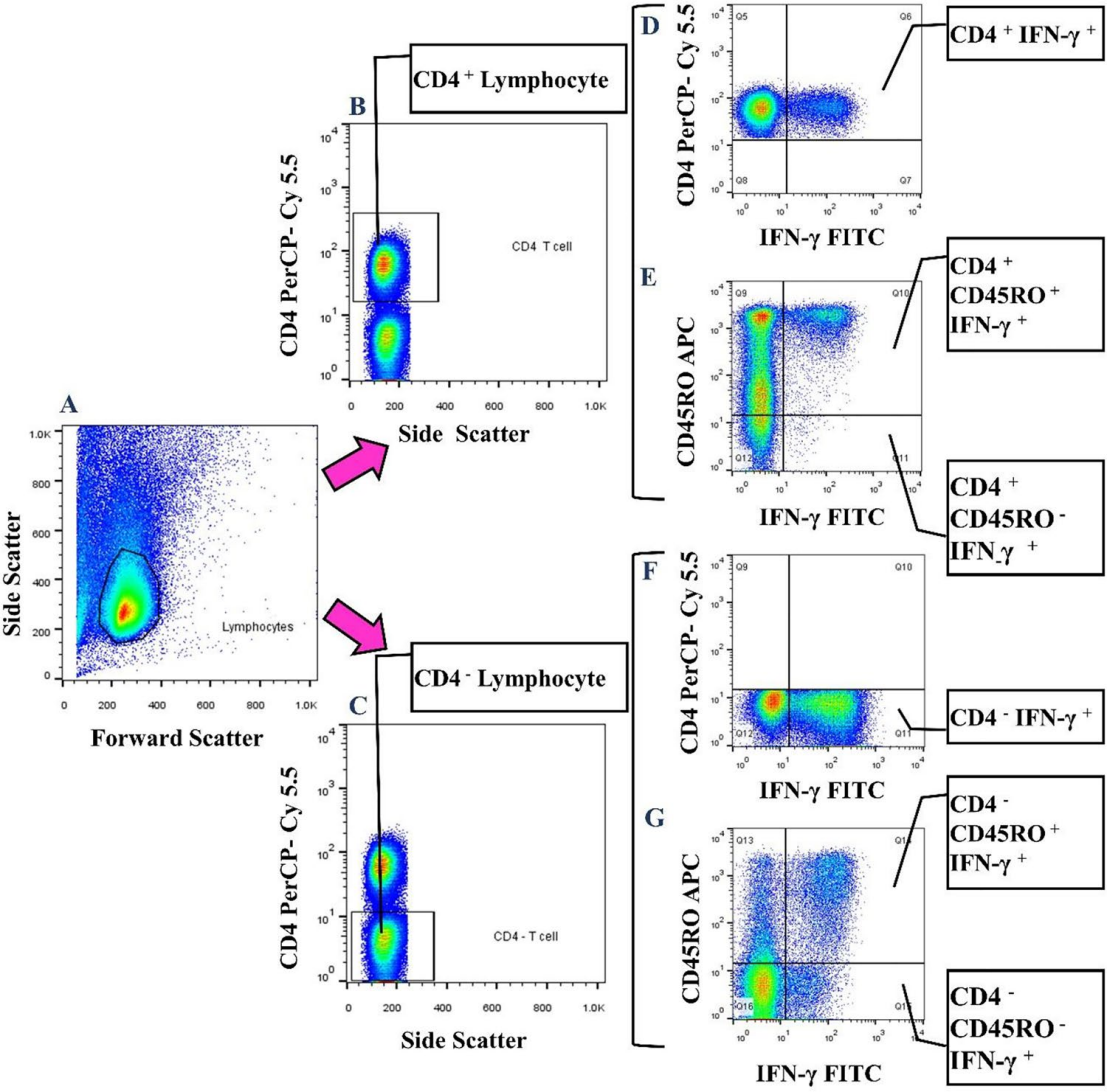
IFN-γ production by CD4^+^ and CD4^−^ memory lymphocytes. After gating total lymphocytes (**A**), CD4^+^ and CD4^−^ lymphocytes were defined (**B**, **C**), and the frequency of different IFN-γ^+^ cells were determined based on the expression of CD45RO and IFN-γ in each population (**D–G**)

To determine the frequency of IFN-γ^+^ memory lymphocytes (CD4^+^CD45RO^+^IFN-γ^+^ and CD4^−^CD45RO^+^IFN-γ^+^) after gating the lymphocyte population-based on their relative size (forward scatter) and granularity (side scatter) (Fig. 3A), CD4^+^ and CD4^−^ lymphocytes were defined (Fig. 3B and C). Then, the frequency of IFN-γ producing cells was determined in each population (Fig. 3D-G).

### Frequency of IFN-γ^+^ memory lymphocytes in responders and nonresponders to HB vaccine

In the second section of the study, the PBMCs were stimulated and the frequencies of different IFN-γ^+^ cells were compared between responders and nonresponders to the HB vaccine. The median (IQ25–75) and mean ± SEM of the frequencies in each group and the *p*-values of their differences were summarized in Table 3. As shown, there were no statistical differences in the frequencies of various IFN-γ producing subsets between responder and nonresponder participants.

**Table 3:** Frequencies of various IFN-γ^+^ lymphocytes in responders and nonresponders to HB vaccine

Cell subset	Nonresponder		Responder		*P*-value
	**Median** **(IQ25–75)**	**Mean ± SEM**	**Median** **(IQ25–75)**	**Mean ± SEM**	
CD4^+^IFNγ^+^	8.82 (4.71–16.6)	11.3 ± 2.11	6.65 (4.65–10.72)	7.38 ± 0.96	0.345
CD4^+^CD45RO^+^IFNγ^+^	7.82 (4.63–16.4)	11.04 ± 2.09	6.55 (4.46–10.04)	7.05 ± 0.92	0.279
CD4^+^CD45RO^−^IFNγ^+^	0.14 (0.08–0.39)	0.25 ± 0.065	0.23 (0.12–0.52)	0.32 ± 0.067	0.311
CD4^−^IFNγ^+^	26.6 (16.46–41.9)	31.01 ± 4.47	26.9 (21.6–29.92)	25.21 ± 2.1	0.945
CD4^−^CD45RO^+^IFNγ^+^	14 (11–26.1)	19.6 ± 3.2	12.2 (11.15–19.5)	13.78 ± 1.42	0.381
CD4^−^CD45RO^−^IFNγ^+^	7.78 (5.6–15.4)	11.36 ± 1.94	10.3 (7.74–15.45)	11.44 ± 1.52	0.534
**Mean expression of IFN-γ in cell subsets (based on MFI)**					
CD4^+^IFNγ^+^	22.42 (16.89–38.87)	26.2 ± 3.5	17.65 (15.62–20.23)	18.68 ± 1.09	0.147
CD4^+^CD45RO^+^IFNγ^+^	24.35 (17.4–38.07)	26.5 ± 3.41	18.22 (16.54–21.48)	19.45 ± 1.23	0.147
CD4^+^CD45RO^−^IFNγ^+^	5.59 (4.06–9.67)	8.76 ± 1.78	4.27 (4.06–5.61)	4.88 ± 0.4	0.134
CD4^−^IFNγ^+^	15.13 (13.9–24.1)	18.6 ± 1.96	15.14 (11.71–17.84)	15.41 ± 1.21	0.369
CD4^−^CD45RO^+^IFNγ^+^	22.24 (15.3–35.8)	26.42 ± 3.94	19.42 (16.21–24.3)	20.66 ± 1.36	0.596
CD4^−^CD45RO^−^IFNγ^+^	10.2 (8.28–13.2)	10.34 ± 0.77	8.33 (7.53–12.02)	9.99 ± 0.88	0.565

*IQ*, interquartile; *SEM*, standard error of mean.

## Discussion

HBV is one of the main reasons for cirrhosis and liver cancer worldwide [2, 8]. Different medications are in clinical use to treat this disease. Because of the challenges during the treatment of hepatitis, vaccination is more efficient as a prophylactic approach [10–13]. The vaccine provides protection via neutralization of the virus by anti-HBsAb and activation of CD4^+^ T memory cells [15]. Although vaccine-induced antibody levels are gradually declining, it has been shown that memory cells are maintained in healthy vaccine recipients for more than 15 years [19–21].

The goal of vaccination is to induce long-lasting protective immune memory [24]. The identification of memory cell subsets may indicate the effectiveness of vaccines. Therefore, we designed the present study to analyze the frequency of CD4^+^ memory T cell subsets (T_SCM_, T_CM_, and T_EM_ cells) and compare these cell quantities between responders and nonresponders to the HB vaccine. These cells were determined based on the differential expression of CCR7, CD45RO, and CD95 markers [23, 26].

Pieces of evidence showed that specific T_SCM_ cells were produced during normal immune responses against pathogens [31–33]. Furthermore, a negative correlation between the severity of disease and the frequency of circulating T_SCM_ cells in chronic viral and parasitic infections was observed [34]. Accordingly, the protective role of T_SCM_ cells makes them an attractive candidate in vaccination and adoptive T cell therapy [34–39]. Our results showed that the nonresponder participants had a lower frequency of CD4^+^CD95^+^, CD4^+^CD95^Hi^, and CD4^+^CD95^low/med^ T_SCM_ than responders to the HB vaccine. However, other subsets, including naïve, central memory, and effector memory CD4^+^ T cells, were not statistically different between the studied groups.

There are some possible mechanisms underlying impaired antibody response to HB vaccination. For instance, several studies revealed a negative correlation between the frequency of regulatory T (Treg) cells and the seroconversion rate after HB vaccination [40, 41]. In addition, decreased frequency and function of monocyte-derived dendritic cells (moDCs) and subsequently attenuated memory T cell induction had been considered a probable reason for being nonresponder to HB vaccine [42]. However, the long-term memory T cell response has not been fully elucidated in this regard [43]. To the best of our knowledge, no similar studies could be found regarding the frequency of various memory T cell subsets, particularly T_SCM_ cells after vaccination, and their comparison between responders and nonresponders of healthy recipients. In the case of vaccination, few studies existed about the induction of T_SCM_ cells following vaccination against yellow fever (YF) and the application of CpG-B based cancer vaccines [44, 45]. In a study by Scriba et al., it was suggested that vaccine-induced T_SCM_ contributed to long-term memory formation and proliferative capacity of the vaccine-induced T cell response to mycobacteria [46]. Moreover, Schlom et al. made a vaccine directed against a transcription factor named Twist that has a role in the metastatic process. They showed an increase in CD4^+^ T_SCM_ cells in vaccinated mice in comparison with PBS-treated mice. This revealed that the T_SCM_ population could generate an antitumor activity [47, 48]. Based on the findings of these studies and our results which showed a lower frequency of T_SCM_ in nonresponders, it can be concluded that the T_SCM_ cells play a crucial role in long-lasting immunological memory response preservation [34]. Also, we assumed that the memory response in nonresponders to the HB vaccine probably had an immunological defect in the memory CD4^+^ T cell formation. Consequently, a lower frequency of T_SCM_ cells might play a principal role in the absence of protection even after several HB vaccine injections in the nonresponder group.

In this study, we also compared the frequency of T_CM_ and T_EM_ between studied groups and observed no statistically significant differences. Previous studies had shown that the frequency of these subsets was correlated with the efficacy of several vaccines like ZOSTAVAX, Influenza, Malaria, and human papillomavirus type 16 (HPV-16) vaccines [49–53]. No study reported the comparison of the frequency of these subsets between responders and nonresponders in the normal population against the HB vaccine. However, some studies investigated these cells after HB vaccination in subjects with a different conditions. For example, Litjens et al. conducted a study on end-stage renal disease (ESRD) patients. They showed that the production of specific CD4^+^ T_EM_ cells was impaired in the patients after administration of the HB vaccine in comparison with healthy controls [54]. Another investigation that was done in a normal population showed a positive association between the frequency and absolute numbers of HBsAg-specific IL-2 producing CD4^+^ T_EM_ cells and HBsAb titer [55]. Moreover, Marchant et al. performed a study on the subjects who received the Engerix-B vaccine. They observed that the HBsAg-specific memory CD4^+^ T cells included both T_CM_ and T_EM_ cells [56]. Two separate studies about HIV-infected individuals who were vaccinated against HBV had shown that the seroconversion stimulated by the vaccine positively correlated with the development of T cell immunological memory [57, 58].

No significant differences observed in our study could be related to stimulation conditions as it was not completely specific because we used HBsAg for stimulation and did not check the specific memory T cells using tetramer staining. In addition, due to the restriction in inclusion criteria of our samples, particularly the nonresponder subjects, the sample size was relatively small. Also, we did not have precise information on the time point of the last vaccination of the subjects of our study, which might have effects on the frequency of memory T cell populations [23].

In this study, we also investigated the correlation between the responders’ age and memory T cell subsets. There was a negative correlation between the age of responders and the frequency of T_N_. Besides, a positive correlation was found between the age of responders and the frequency of CD45RO^+^CD95^+^ T_CM_ and CD45RO^low/med^CD95^+^ T_CM_. There were similar studies that examined the correlation between the age of healthy individuals and the frequency of different memory T cell subsets [59–62]. Based on our findings and other studies, it can be implied that the turnover and long lifespan of T_N_ cells are decreasing during aging, probably due to a decline in thymic output and/or depletion of naïve repertoire by activation. Accordingly, the thymus becomes unable to substitute the lost T_N_ cells in the periphery. In contrast to T_N_, cumulative exposure to foreign pathogens and environmental antigens induces the accumulation of memory T cells with aging, which is in line with our study [63].

Several studies focus on IFN-γ production as an indicator of cellular immunity, and some of them have identified a clear dominance of the T helper (Th)1 phenotype after HB vaccination. Moreover, a strong correlation has been shown between the HBsAg-specific IFN-γ^+^ T cell response and HBsAb level. In the last part of our study, we evaluated the frequency of memory T cells secreting IFN-γ in studied groups. In agreement with Makhlouf et al. study, memory CD4^+^ T cells secreting IFN-γ were detectable after in vitro activation by HBsAg in both responder and nonresponder individuals. Although, Makhlouf’s study showed that the percentage of CD4^+^CD45RO^+^IFN-γ^+^ memory T cells was significantly higher in the responder participants than in nonresponders, we did not find any statistical differences neither in the frequency nor in the mean expression of IFN-γ in the CD4^+^CD45RO^+^IFN-γ^+^ memory T cells.

The limitations mentioned earlier, including nonspecific detection of memory T cells, small sample size, and the uncertain time interval between sampling and vaccination, are likely to affect our results regarding memory T cells secreting IFN-γ. Most likely, using more specific methods for detection of memory T cells against HBsAg producing IFN-γ should be taken into consideration in future studies. Also, the interval of the last vaccine administration and the time of investigations must be kept in mind.

In conclusion, based on our observations, it is likely that nonresponders to the HB vaccine have a defect in their immunological memory CD4^+^ T cell formation due to their lower frequency of T_SCM_ cells compared to the responders. It may play an important role in lower anti-HBsAb production after HB vaccination in nonresponders. For further research, using recombinant HB vaccine containing safe adjuvants, administration of a vaccine with different intervals and doses of injections in nonresponders may increase the frequency of memory T cell subsets, especially T_SCM_ cells.

## Supplementary Information

Below is the link to the electronic supplementary material.

Supplementary file1 (DOCX 25 KB)
