# Supplementary material for: Lower frequency of T stem cell memory (TSCM) cells in hepatitis B vaccine nonresponders
Source: Immunol Res. 2022 Apr 20;70(4):469–80. doi: 10.1007/s12026-022-09278-9 (PMC9273562; doi:10.1007/s12026-022-09278-9)
Supplement: Supplementary file 1 — Supplementary file1 (DOCX 25 KB) [file 12026_2022_9278_MOESM1_ESM.docx]

Table S1 The statistical parameters of the frequency of different CD4^+^ memory T cell subsets in responder group.

| **Cell subset** | **Markers** | **Min** | **Max** | **Median**  **(IQ25-75)** | **Mean ± SEM** |
| --- | --- | --- | --- | --- | --- |
| **CD4^+^ T** | CD4^+^ | 20.6 | 62.9 | 39.9  (37.7-52.95) | 44.07±3.09 |
| **CD4^+^ CD45RO^+^** | CD4^+^ CD45RO^+^ | 59.1 | 85.3 | 75.1  (69.95-82.1) | 74.91±2.18 |
|  | CD4^+^ CD45RO^Hi^ | 13.2 | 43.9 | 28.8  (22.25-35.35) | 29.22±2.43 |
|  | CD4^+^ CD45RO^Low/Med^ | 31.6 | 55.3 | 46.5  (40.5-50) | 45.69±1.88 |
| **CD4^+^ CD45RO^-^** | CD4^+^ CD45RO^-^ | 14.7 | 40.9 | 24.9  (17.9-30.05) | 25.08±2.18 |
| **CD4^+^ CCR7^+^** | CD4^+^ CCR7^+^ | 91.6 | 98.6 | 97  (94.05-97.6) | 96±0.59 |
| **CD4^+^ CCR7^-^** | CD4^+^ CCR7^-^ | 1.4 | 8.4 | 3  (2.4-5.95) | 3.99±0.59 |
| **T_N_** | CD4^+^ CCR7^+^CD45RO^-^ CD95^-^ | 1.79 | 13.91 | 3.92  (3.08-7.18) | 5.46±0.92 |
| **T_SCM_** | CD4^+^ CCR7^+^CD45RO^-^ CD95^+^ | 11.9 | 31.7 | 19.01  (13.78-23.07) | 19.59±1.78 |
|  | CD4^+^ CCR7^+^CD45RO^-^ CD95^Hi^ | 0.96 | 3.2 | 1.87  (1.46-2.47) | 2.02±0.19 |
|  | CD4^+^ CCR7^+^CD45RO^-^ CD95^Low/Med^ | 10.4 | 29.2 | 17.13  (12.17-20.36) | 17.57±1.63 |
| **CD45RO^+^ T_CM_** | CD4^+^ CCR7^+^CD45RO^+^ CD95^+^ | 53.95 | 80.62 | 66.28  (56.19-71.92) | 65.73±2.47 |
|  | CD4^+^ CCR7^+^CD45RO^+^ CD95^Hi^ | 25.37 | 57.58 | 36.58  (31.37-39.72) | 37.09±2.19 |
|  | CD4^+^ CCR7^+^CD45RO^+^ CD95 ^Low/Med^ | 17.66 | 44.51 | 26.23  (21.74-36.32) | 28.64±2.39 |
| **CD45RO^Hi^ T_CM_** | CD4^+^ CCR7^+^CD45RO^Hi^ CD95^+^ | 10.69 | 43.5 | 27.8  (21.34-33.58) | 27.61±2.52 |
|  | CD4^+^ CCR7^+^CD45RO^Hi^ CD95^Hi^ | 9.61 | 41.54 | 26.33  (17.35-27.73) | 24.63±2.45 |
|  | CD4^+^ CCR7^+^CD45RO^Hi^ CD95 ^Low/Med^ | 0.5 | 9.59 | 2.11  (1.12-4.42) | 2.98±.071 |
| **CD45RO ^Low/Med^ T_CM_** | CD4^+^ CCR7^+^CD45RO ^Low/Med^ CD95^+^ | 26.16 | 50.46 | 36.05  (30.73-46.1) | 38.05±2.36 |
|  | CD4^+^ CCR7^+^CD45RO ^Low/Med^ CD95^Hi^ | 6.13 | 18.52 | 12.66  (10.39-15.55) | 12.64±0.93 |
|  | CD4^+^ CCR7^+^CD45RO ^Low/Med^ CD95 ^Low/Med^ | 13.16 | 38.54 | 24.41  (19.68-32.6) | 25.41±2.09 |
| **T_EM_** | CD4^+^ CCR7^-^CD45RO^+^ CD95^+^ | 1.38 | 8.27 | 2.96  (2.33-5.91) | 3.9±0.59 |
|  | CD4^+^ CCR7^-^CD45RO^+^ CD95^Hi^ | 0.52 | 7.1 | 2.8  (1.47-4.31) | 2.96±0.5 |
|  | CD4^+^ CCR7^-^CD45RO^+^ CD95 ^Low/Med^ | 0.01 | 4.57 | 0.68  (0.16-1.13) | 0.94±0.34 |
| **T_TE_** | CD4^+^ CCR7^-^CD45RO^-^ | 0 | 0.6 | 0.01  (0-0.04) | 0.06±0.04 |
| **Mean expression of CD95 on CD4^+^ T_SCM_ cell subsets (based on MFI)** | | | | | |
| **CD95^+^ T_SCM_** | CD4^+^ CCR7^+^CD45RO^-^ CD95^+^ | 2.84 | 6.74 | 4.48  (3.5-5.7) | 4.47±0.34 |
| **CD95^Hi^ T_SCM_** | CD4^+^ CCR7^+^CD45RO^-^ CD95^Hi^ | 7.89 | 23.3 | 11.96  (9.56-19.68) | 14.05±1.56 |

Min: Minimum; Max: Maximum; IQ: Interquartile; SEM: Standard Error of Mean.

**Table S2** The statistical parameters of the frequency of different CD4^+^ memory T cell subsets in non-responder group**.**

| **Cell subset** | **Markers** | **Min** | **Max** | **Median**  **(IQ25-75)** | **Mean ± SEM** |
| --- | --- | --- | --- | --- | --- |
| **CD4^+^ T** | CD4^+^ | 23.5 | 60.6 | 40.8  (33.4-50.7) | 41.3±2.6 |
| **CD4^+^ CD45RO^+^** | CD4^+^ CD45RO^+^ | 66.6 | 89.8 | 82.8  (74.6-88.8) | 80.76±2.15 |
|  | CD4^+^ CD45RO^Hi^ | 19 | 61.5 | 39.1  (27-48.3) | 38.1±3.26 |
|  | CD4^+^ CD45RO ^Low/Med^ | 28.3 | 58 | 44.9  (34.5-47.5) | 42.66±1.99 |
| **CD4^+^ CD45RO^-^** | CD4^+^ CD45RO^-^ | 10.2 | 33.4 | 17.2  (11.2-25.4) | 19.24±2.15 |
| **CD4^+^ CCR7^+^** | CD4^+^ CCR7^+^ | 77.9 | 98.7 | 94.6  (90.6-96.7) | 93.02±1.43 |
| **CD4^+^ CCR7^-^** | CD4^+^ CCR7^-^ | 1.3 | 22.1 | 5.4  (3.3-9.4) | 6.98±1.43 |
| **T_N_** | CD4^+^ CCR7^+^CD45RO^-^ CD95^-^ | 2.28 | 15.72 | 5.23  (3.09-7.36) | 5.79±0.92 |
| **T_SCM_** | CD4^+^ CCR7^+^CD45RO^-^ CD95^+^ | 5.76 | 23.58 | 12.92  (8-17.84) | 13.37±1.45 |
|  | CD4^+^ CCR7^+^CD45RO^-^ CD95^Hi^ | 0.52 | 2.6 | 1.05  (0.67-1.39) | 1.08±0.14 |
|  | CD4^+^ CCR7^+^CD45RO^-^ CD95 ^Low/Med^ | 5.08 | 22.02 | 12.07  (7.37-16.63) | 12.28±1.34 |
| **CD45RO^+^ T_CM_** | CD4^+^ CCR7^+^CD45RO^+^ CD95^+^ | 52.27 | 80.66 | 68.25  (59.44-76.44) | 67.93±2.37 |
|  | CD4^+^ CCR7^+^CD45RO^+^ CD95^Hi^ | 25.52 | 57.98 | 38.25  (31.26-47.75) | 40.38±2.48 |
|  | CD4^+^ CCR7^+^CD45RO^+^ CD95 ^Low/Med^ | 14.7 | 45.18 | 23.98  (19.84-36.63) | 27.55±2.58 |
| **CD45RO^Hi^ T_CM_** | CD4^+^ CCR7^+^CD45RO^Hi^ CD95^+^ | 18.76 | 45.65 | 30.9  (22.68-42.78) | 32.75±2.61 |
|  | CD4^+^ CCR7^+^CD45RO^Hi^ CD95^Hi^ | 16.8 | 41.37 | 26.75  (19.98-39.32) | 28.26±2.34 |
|  | CD4^+^ CCR7^+^CD45RO^Hi^ CD95 ^Low/Med^ | 0.15 | 18.9 | 2.7  (1.14-5.45) | 4.49±1.3 |
| **CD45RO ^Low/Med^ T_CM_** | CD4^+^ CCR7^+^CD45RO ^Low/Med^ CD95^+^ | 22.2 | 47.73 | 36.04  (29.52-41.77) | 35.64±1.83 |
|  | CD4^+^ CCR7^+^CD45RO ^Low/Med^ CD95^Hi^ | 6.9 | 18.29 | 12.38  (9.1-14.95) | 12.25±0.92 |
|  | CD4^+^ CCR7^+^CD45RO ^Low/Med^ CD95 ^Low/Med^ | 13.1 | 36.41 | 22.99  (19.28-27.94) | 23.39±1.89 |
| **T_EM_** | CD4^+^ CCR7^-^CD45RO^+^ CD95^+^ | 1.3 | 21.6 | 5.38  (3.3-8.9) | 6.8±1.4 |
|  | CD4^+^ CCR7^-^CD45RO^+^ CD95^Hi^ | 0.86 | 20.8 | 3.62  (1.95-5.97) | 5.43±1.4 |
|  | CD4^+^ CCR7^-^CD45RO^+^ CD95 ^Low/Med^ | 0.05 | 4.9 | 0.8  (0.36-2.18) | 1.37±0.38 |
| **T_TE_** | CD4^+^ CCR7^-^CD45RO^-^ | 0 | 0.5 | 0.01  (0-0.09) | 0.09±0.04 |
| **Mean expression of CD95 on CD4^+^ T_SCM_ cell subsets (based on MFI)** | | | | | |
| **CD95^+^ T_SCM_** | CD4^+^ CCR7^+^CD45RO^-^ CD95^+^ | 2.37 | 12.47 | 3.41  (3.1-5.29) | 4.4±0.65 |
| **CD95^Hi^ T_SCM_** | CD4^+^ CCR7^+^CD45RO^-^ CD95^Hi^ | 5.52 | 63.23 | 11.31  (8.43-21.28) | 16.06±3.72 |

Min: Minimum; Max: Maximum; IQ: Interquartile; SEM: Standard Error of Mean.

Table S3 The correlation of anti-HBsAb level and the age of participants with the frequency of different cell subsets in responder and non-responder group.

| **Cell subsets** | **Non-responder** | | **responder** | |
| --- | --- | --- | --- | --- |
|  | **Age** | **Ab titer** | **Age** | **Ab titer** |
| **CD4^+^ CD45RO^+^** | p-value:  0.25 | p-value:  0.44 | p-value:  0.09 | p-value:  0.7 |
| **T_N_** | p-value:  0.72 | p-value:  0.25 | **p-value:**  **0.029**  **CC: - 0.6** | p-value:  0.57 |
| **CD95^+^ T_SCM_** | p-value:  0.34 | p-value:  0.43 | p-value:  0.63 | p-value:  0.76 |
| **CD95^Hi^ T_SCM_** | p-value:  0.84 | p-value:  0.29 | p-value:  0.38 | p-value:  0.86 |
| **CD95^+^ CD45RO^+^ T_CM_** | p-value:  0.56 | p-value:  0.62 | **p-value:**  **0.043**  **CC: 0.57** | p-value:  0.62 |
| **CD95^Hi^ CD45RO^+^ T_CM_** | p-value:  0.46 | p-value:  0.94 | p-value:  0.45 | p-value:  0.37 |
| **CD95^+^ CD45RO^Hi^ T_CM_** | p-value:  0.99 | p-value:  0.59 | p-value:  0.56 | p-value:  0.32 |
| **CD95^Hi^ CD45RO^Hi^ T_CM_** | p-value:  0.66 | p-value:  0.61 | p-value:  0.29 | p-value:  0.31 |
| **CD95^+^ CD45RO^Low/Med^ T_CM_** | p-value:  0.58 | p-value:  0.76 | **p-value:**  **0.014**  **CC: 0.66** | p-value:  0.2 |
| **CD95^Hi^ CD45RO^Low/Med^ T_CM_** | p-value:  0.13 | p-value:  0.12 | p-value:  0.67 | p-value:  0.84 |
| **CD95^+^ T_EM_** | p-value:  0.57 | p-value:  0.58 | p-value:  0.73 | p-value:  0.47 |
| **CD95^Hi^ T_EM_** | p-value:  0.88 | p-value:  0.46 | p-value:  0.15 | p-value:  0.73 |

CC: correlation coefficient.
